# The Characterisation of an Alkali-Stable Maltogenic Amylase from *Bacillus lehensis* G1 and Improved Malto-Oligosaccharide Production by Hydrolysis Suppression

**DOI:** 10.1371/journal.pone.0106481

**Published:** 2014-09-15

**Authors:** Nor Hasmaliana Abdul Manas, Samson Pachelles, Nor Muhammad Mahadi, Rosli Md. Illias

**Affiliations:** 1 Department of Bioprocess Engineering, Faculty of Chemical Engineering, Universiti Teknologi Malaysia, Skudai, Johor, Malaysia; 2 Comparative Genomics and Genetics Research Centre, Malaysia Genome Institute, Kajang, Selangor, Malaysia; University of Insubria, Italy

## Abstract

A maltogenic amylase (MAG1) from alkaliphilic *Bacillus lehensis* G1 was cloned, expressed in *Escherichia coli*, purified and characterised for its hydrolysis and transglycosylation properties. The enzyme exhibited high stability at pH values from 7.0 to 10.0. The hydrolysis of β-cyclodextrin (β-CD) produced malto-oligosaccharides of various lengths. In addition to hydrolysis, MAG1 also demonstrated transglycosylation activity for the synthesis of longer malto-oligosaccharides. The thermodynamic equilibrium of the multiple reactions was shifted towards synthesis when the reaction conditions were optimised and the water activity was suppressed, which resulted in a yield of 38% transglycosylation products consisting of malto-oligosaccharides of various lengths. Thin layer chromatography and high-performance liquid chromatography analyses revealed the presence of malto-oligosaccharides with a higher degree of polymerisation than maltoheptaose, which has never been reported for other maltogenic amylases. The addition of organic solvents into the reaction further suppressed the water activity. The increase in the transglycosylation-to-hydrolysis ratio from 1.29 to 2.15 and the increased specificity toward maltopentaose production demonstrated the enhanced synthetic property of the enzyme. The high transglycosylation activity of maltogenic amylase offers a great advantage for synthesising malto-oligosaccharides and rare carbohydrates.

## Introduction

Oligosaccharides offer numerous health benefits and improve the physicochemical properties of foods [1]. A progressive increase in the demand for oligosaccharides has increased the need for efficient catalysts. Oligosaccharides can be synthesised chemically, but enzymatic synthesis has generally been the first choice because it employs milder conditions, involves simpler steps and eliminates the need for hydroxyl group protection [2]. Glycosyl transferases (EC 2.4) and glycosyl hydrolases (EC 3.2) are the two candidate enzyme classes that have been used in oligosaccharides synthesis [3]. However, glycosyl hydrolases are preferred because these enzymes can use simple and inexpensive acceptor sugar molecules [2].

Maltogenic amylase (EC 3.2.1.133) is a potential catalyst for malto-oligosaccharide production. It is an amylolytic enzyme from glycosyl hydrolase family 13 (GH13). Maltogenic amylase, pullulanase (EC 3.2.1.4), cyclodextrin glucanotransferase (EC 2.4.1.19) and cyclodextrinase (EC 3.2.1.54) are known to exhibit different substrate specificities compared with α-amylase (EC 2.3.1.1), even though they are from the same family. These enzymes allow for a reaction with multiple substrates, such as starch, pullulan and cyclodextrins [4]. The cyclodextrins are preferred as a substrate, in contrast with the typical α-amylases, which hydrolyse starch more efficiently. Maltogenic amylase has been demonstrated to have a transglycosylation activity that forms sugar molecules of various lengths [5]. This enzyme reportedly catalysed the formation of glycosidic linkages to produce oligosaccharides and various modified sugars [6], [7], [8]. The efficient production of isomalto-oligosaccharides from liquefied starch using maltogenic amylase has also been reported [9], [10].

Although glycosyl hydrolases are preferred for oligosaccharide production, their major shortcoming is the inevitable hydrolysis activity that causes synthesised oligosaccharides to be hydrolysed again by the enzyme [11]. Various strategies have been employed to overcome the problem, including the elimination of water, which is a competing nucleophile, for transglycosylation. The incorporation of an organic medium into the reaction mixture has increased the synthesis of galacto-oligosaccharides by β-glycosidase [12]. However, no study on the influence of organic solvents on malto-oligosaccharide synthesis by maltogenic amylase has been reported to date.

The present article reports the cloning, expression, purification and characterisation of a maltogenic amylase (MAG1) from locally isolated *Bacillus lehensis* G1. The products of β-CD hydrolysis and malto-oligosaccharide synthesis by transglycosylation were observed. The optimisation of transglycosylation conditions was performed to minimise the accompanying hydrolysis reaction. The effect of an organic solvent in improving the transglycosylation-to-hydrolysis ratio was first reported for maltogenic amylase. This report is also the first to describe the optimisation of reaction conditions and the incorporation of a water-miscible organic solvent to suppress hydrolysis activity during malto-oligosaccharide production by maltogenic amylase. The results presented here suggest that MAG1 is a promising candidate to catalyse the production of malto-oligosaccharides of various lengths.

## Materials and Methods

### Bacterial strains, plasmids and media

The alkaliphilic bacterium *B. lehensis* G1 was grown in Horikoshi Broth (HB). *Escherichia coli* JM109 and *E. coli* BL21 (DE3) (Promega, Madison, WI, USA) were used as a cloning host and expression host, respectively, in the present study. pET21a (+) from Novagen (Merck KGaA, Darmstadt, Germany) was used as an expression plasmid. *E. coli* strains harbouring the recombinant plasmid were grown in Luria-Bertani (LB) media supplemented, when necessary, with 100 µg/ml ampicillin.

### DNA manipulation and cloning

The extraction of genomic and plasmid DNA, restriction enzyme digestion, ligation, DNA separation by agarose gel electrophoresis and transformation were performed as described by Sambrook [13]. The gene encoding maltogenic amylase was amplified from the *B. lehensis* G1 genome by polymerase chain reaction (PCR) with KOD Hot Start DNA Polymerase from Novagen. The following primers were used for gene amplification: MA-F (5′CCG GGG ATC CAT GAA TCG AGC TGG CAT TAT T3′) and MA-R (5′GGC CCT CGA GTT ATG ATG TGA GCT TTA ATT G3′). The cycling conditions were 95°C for 2 min followed by 30 cycles of 95°C for 30 s, 60°C for 30 s, and 70°C for 1 min, with a final extension at 70°C for 5 min. Both the PCR product and plasmid were subjected to restriction enzyme digestion before their ligation and transformation into the *E. coli* host. The DNA modification was verified by DNA sequencing (Genomics Bioscience and Technology Sdn Bhd, Kuala Lumpur, Malaysia).

### Enzyme purification, SDS-PAGE and identification of the oligomeric form

The MAG1 gene was expressed in *E. coli* BL21 (DE3) under the control of the T7 promoter in pET21a (+) by inducing with 0.5 mM isopropyl β-_D_-1-thiogalactopyranoside (IPTG). The recombinant *E. coli* was grown in LB medium supplemented with 100 µg/ml ampicillin at 30°C with shaking for 12 hours. The cells were harvested by centrifugation at 8,000×*g* for 20 min. The cell pellets were suspended in 50 mM potassium phosphate buffer pH 7.0 followed by sonication in an ice bath. The cell debris was removed by centrifugation at 10,000×*g* for 30 min at 4°C. The supernatant constituting the enzyme crude extract was injected into a nickel-nitrilotriacetic acid (Ni-NTA) column with an AKTAPrime Plus Purification System (GE Healthcare, Little Chalfont, Buckinghamshire, UK). Unbound proteins were removed by washing with phosphate-buffered saline (20 mM Na_2_HPO_4_ and 500 mM NaCl, pH 7.4). The protein was eluted by gradient elution with the same buffer containing 500 mM imidazole. All of the procedures described were performed at 4°C.

The protein was observed by SDS-PAGE [14] in 12% polyacrylamide gels. Western blotting was performed with His•Tag AP Western 87 Reagents Kits from Novagen with a his•tag monoclonal antibody and a goat anti-mouse IgG AP conjugate. The oligomeric form was determined by subjecting the purified protein to gel filtration chromatography with a HiLoad Superdex 200 13/300 column (GE Healthcare) in 20 mM phosphate buffer and 150 mM NaCl. The molecular weight was estimated from a calibration curve that was prepared by using the standard proteins ferritin (440 kDa), aldolase (158 kDa), conalbumin (75 kDa), ovalbumin (44 kDa), carbonic anhydrase (29 kDa), ribonuclease A (13.7 kDa) and aprotinin (6.5 kDa).

### Enzyme assay and protein assay

The MAG1 activity was measured by the dinitrosalicylic acid (DNS) method [15] using β-CD as a substrate in 50 mM potassium phosphate buffer, pH 7.0, at 40°C. One unit of enzyme activity was defined as the amount of enzyme required to produce 1 µmol of maltose per min under optimal conditions. The protein concentration was measured by the Bradford method [16] with bovine serine albumin as the standard.

### Biochemical characterisation, substrate specificity and enzyme kinetics

The optimum activity for the hydrolysis activity was determined by incubating the reaction mixtures (pH 7.0) at 5°C to 80°C. The optimum pH was determined by incubating the reaction mixtures at 40°C in buffers of varying pH (4.0 to 10.0). The thermostability was evaluated by pre-incubating the enzyme at different temperatures (10°C to 60°C) for 10 min. The residual enzymatic activity was subsequently measured by the DNS method. The enzyme's stability with respect to pH was evaluated by pre-incubating the enzymes in buffers with different pH values (3.0 to 11.0) at 4°C for 30 min and followed by the DNS method for determination of the residual activity. The thermal inactivation of MAG1 was analysed by assaying the residual activity at the desired intervals after incubating the enzyme at different temperatures (−20°C to 40°C). Following the first-order kinetics, ln[residual activity] versus time was plotted. The half-life of thermal inactivation, T_1/2_, was calculated as ln2/K_d_, where the deactivation rate constant, K_d_, of irreversible thermal denaturation was obtained from the slope of the plot [17].

The effects of metal ions (K^+^, Ca^2+^, Co^2+^, Fe^2+^, Mg^2+^, Mn^2+^, Zn^2+^, Cu^2+^, Li^2+^, Pb^2+^ and Ni^2+^) and additives (2-mercaptoethanol (2-ME), ethylenediaminetetraacetic acid (EDTA), phenylmethylsulfonyl fluoride (PMSF), sodium dodecyl sulphate (SDS) and Tween 20) on the enzyme activity were determined by adding the reagents to the assay reaction mixture at concentrations of 5 mM for metal ions and 1% (v/v) for additives. The substrate specificity was determined by conducting the assay with different substrates (α-CD, β-CD, γ-CD, amylose, amylopectin and starch). The kinetic parameters (V_max_, K_m_, K_cat_ and K_cat_/K_m_) were calculated from a Lineweaver-Burk plot.

### The hydrolysis and transglycosylation activity of MAG1

For the time course analysis of β-CD hydrolysis, MAG1 was mixed with 0.5% (w/v) β-CD in 50 mM potassium phosphate buffer, pH 7.0 and incubated at 40°C for 6 hours. Aliquots were drawn out at the specified intervals and analysed by thin layer chromatography (TLC) and high-performance liquid chromatography (HPLC). MAG1 was incubated with 5% (w/v) malto-oligosaccharides to determine the best acceptor for transglycosylation. The optimisation of the transglycosylation was performed by incubating MAG1 with maltotriose, and the reaction parameters were manipulated accordingly. The reaction was stopped by boiling for 5 min and was analysed by TLC and HPLC.

### Thin layer chromatography

A silica gel TLC plate (Merck) was activated by placing it on a 110°C hot plate for 30 min. The samples and standards were spotted on the bottom of the TLC plate, left to dry and then developed with a mobile phase (isopropyl alcohol:ethyl acetate:water, 3∶1∶1 (v/v/v)) in a TLC chamber at room temperature. The plate was dried and dipped rapidly into a solution containing 5% (v/v) concentrated H_2_SO_4_ in methanol. The plate was dried and placed on a 110°C hot plate until the spots appeared.

### High-performance liquid chromatography

The samples were filtered through a 0.2-µm-pore-sized syringe filter prior to injection into the HPLC system (Waters, Milford, MA, USA). An RSO oligosaccharide column (Phenomenex, Torrance, CA, USA) was employed to analyse the hydrolysis and transglycosylation products using deionised water as the mobile phase. The elution was monitored using a refractive index (RI) detector.

### Nucleotide sequence accession number

The nucleotide sequence of MAG1 has been deposited in the GenBank database under the accession number KJ416416.

## Results and Discussion

### Maltogenic amylase (MAG1) from *B. lehensis* G1

**Figure 1:**
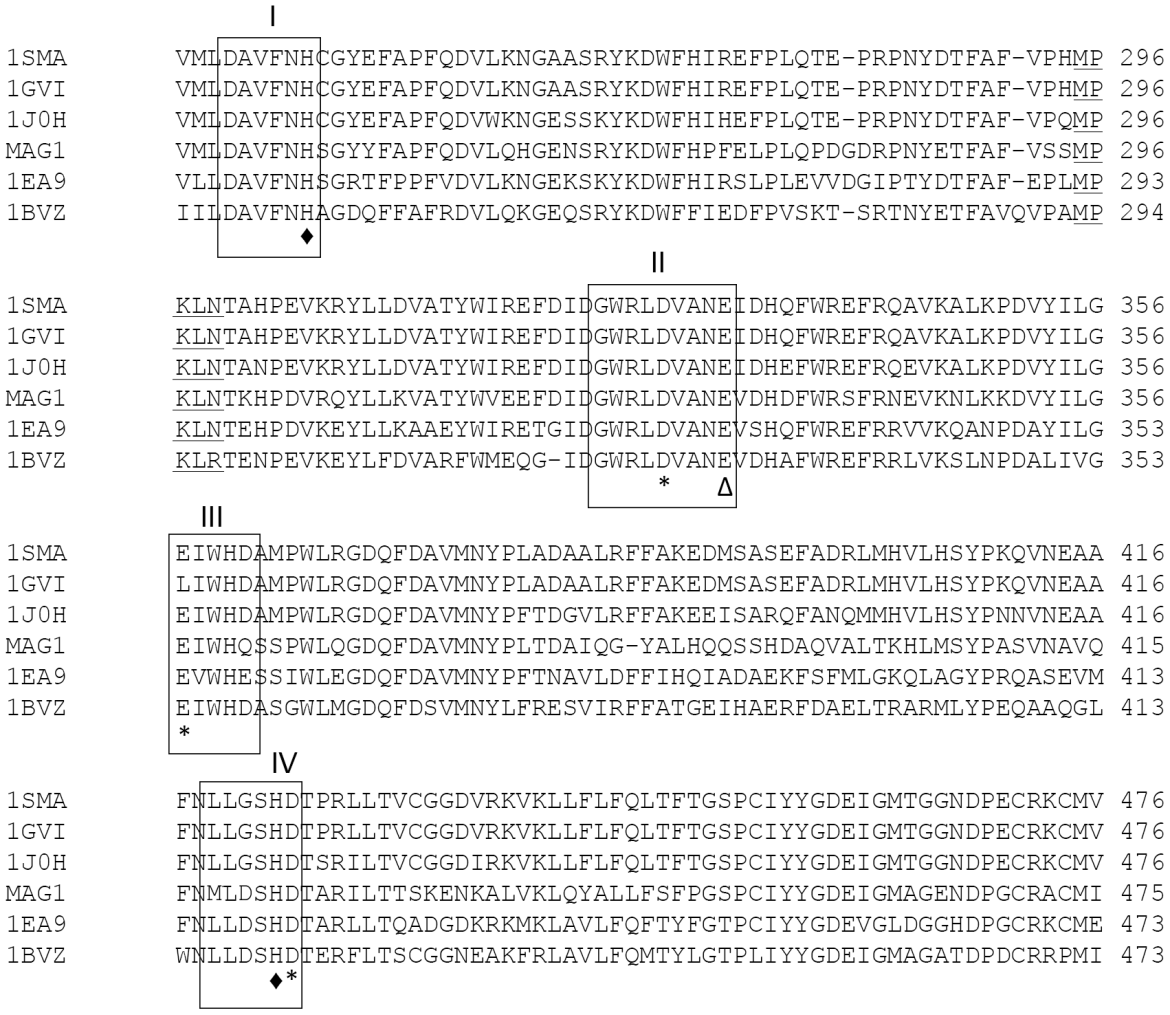
A multiple sequence alignment of the active site centre region of MAG1 with its homologous enzymes. 1SMA, maltogenic amylase from *Thermus* sp. IM6501; 1GVI, maltogenic amylase from *Thermus* sp.; 1J0H, neopullulanase from *Bacillus stearothermophilus*; MAG1, maltogenic amylase from *Bacillus lehensis* G1; 1EA9, cyclomaltodextrinase from *Bacillus* sp.; and 1BVZ, α-amylase II from *Thermoactinomyces vulgaris* R-47. The sequences in the numbered boxes are the conserved regions. The conserved region in most CD-degrading enzymes is underlined. The residues marked with (*), (♦) and (Δ) are the catalytic residues, substrate-binding residues and transglycosylation residues respectively.

The gene sequence that corresponded to MAG1 consisted of 1,746 nucleotides that encoded a protein of 582 amino acid residues. According to a BLAST search [18], the encoded protein exhibited identities with other amylolytic enzymes belonging to the GH13 family. MAG1 shared the highest percentage of amino acid identity with maltogenic amylase from *Thermus* sp. IM6501 (55%), followed by maltogenic amylase from *Thermus* sp. (55%), neopullulanase from *Bacillus stearothermophilus* (54%), cyclomaltodextrinase from *Bacillus* sp. (48%) and α-amylase II from *Thermoactinomyces vulgaris* R-47 (42%). A multiple sequence alignment performed with ClustalW [19] revealed that the four highly important regions among enzymes in the GH13 family were conserved in MAG1, as were the two binding residues and three catalytic residues (Figure 1).

### The oligomeric form and characterisation of MAG1

**Figure 2:**
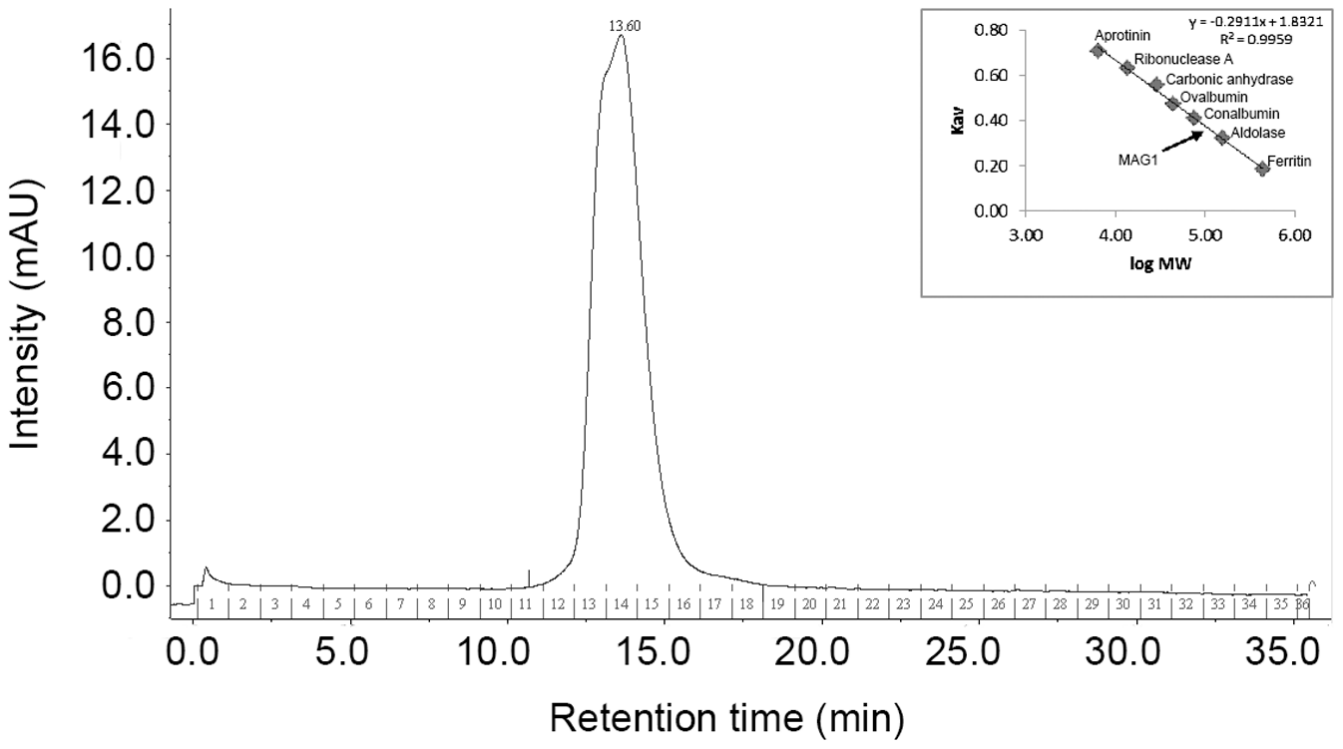
The elution profile of MAG1 by gel filtration chromatography for the determination of the oligomeric form. The standard curve K_av_ versus log MW graph was plotted by using protein standards of known molecular masses.

The recombinant MAG1 was purified to homogeneity from an *E. coli* BL21 (DE3) cell extract by nickel affinity chromatography with an 85% recovery and a 3-fold increase in the specific activity. The protein resolved as a single band at the expected size of 68,000 Da in SDS-PAGE and western blotting (not shown). The estimated molecular mass of MAG1 from the gel filtration chromatography was approximately 104,713 Da (Figure 2). The elution profile exhibited the presence of intermediate molecules; these molecules existed as a mixture of both monomeric and dimeric forms and thus eluted at an intermediate molecular weight [20].

**Figure 3:**
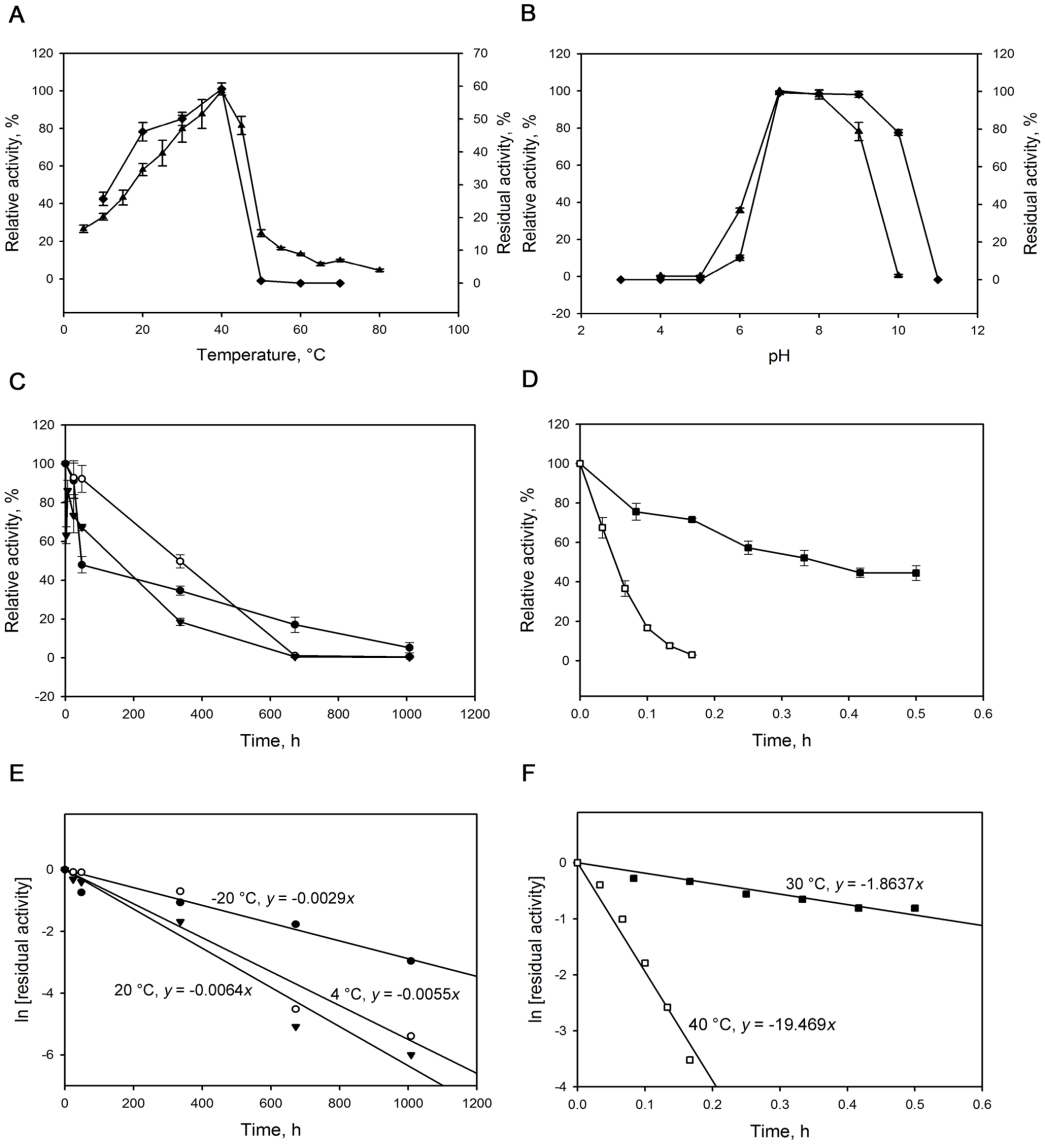
Biochemical characterisation of MAG1. (A) The effects of temperature on MAG1 activity (▴) and stability (♦); (B) The effects of pH on MAG1 activity (▴) and stability (♦); (C) Temperature stability of MAG1 at −20°C (•), 4°C (○) and 20°C (▾); (D) Temperature stability of MAG1 at 30°C (▪) and 40°C (□); (E) Plot of ln[residual activity] vs time (h) for temperature -20°C (•), 4°C (○) and 20°C (▾); (F) Plot of ln[residual activity] vs time (h) for temperature 30°C (▪) and 40°C (□). The error bars represent the standard deviations of triplicate experiments.

MAG1 was characterised as a typical mesophilic enzyme as demonstrated by the low optimal temperature and the lack of thermal stability. As shown in Figure 3A, the enzyme exhibited optimum activity at 40°C. Maltogenic amylases originating from the mesophilic *Bacillus subtilis* SUH4-2 and *Bacillus* sp. US149 also responded to the same optimum temperature [20], [21]. MAG1 could retain more than 50% of its activity at 40°C and 30°C for 10 min. The enzyme was highly active and stable in a pH range from 7.0 to 9.0 with an optimum pH of 7.0 (Figure 3B). A similar optimum pH was recorded for maltogenic amylase from *Geobacillus caldoxylosilyticus* TK4 [22]. MAG1 was also capable of retaining approximately 80% of its activity after 30 min of incubation at pH 10.0 (Figure 3B). Having originated from an alkaliphilic bacterium, MAG1 was expected to display high stability in an alkaline environment. A recombinant maltogenic amylase from *Staphylothermus marinus* (SMMA) [23] is the only maltogenic amylase other than MAG1 that has reportedly exhibited high stability at pH 10.0.

The thermal inactivation analysis revealed that the deactivation of MAG1 occurred more slowly when the enzyme was kept at lower temperatures (-20°C, 4°C and 20°C) than at higher temperatures (30°C and 40°C), as demonstrated in Figure 3C and 3D. The first-order kinetics of thermal inactivation were obeyed, as the residual activity was proportional to the rate of deactivation. Figures 3E and 3F show that the slope (K_d_) increased as the temperature increased. As summarised in Table 1, the half-lives of MAG1 were 239.02 h, 126.03 h and 108.30 h at −20°C, 4°C and 20°C, respectively. However, the half-lives were dramatically reduced to 0.37 h and 0.04 h at 30°C and 40°C, respectively. The significant decline in the half-lives at higher temperature was predicted as MAG1 originated from a mesophilic bacterium. Nevertheless, MAG1 was very stable at temperatures of 20°C and below, which shows that MAG1 is not susceptible to rapid degradation during long-term storage.

**Table 1:** The thermal deactivation constants (K_d_) and half-lives (T_1/2_) values of MAG1 at various temperatures.

Temperature (°C)	K_d_ (h^-1^)	T_1/2_ (h)
−20	0.0029	239.02
4	0.0055	126.03
20	0.0064	108.30
30	1.8637	0.37
40	19.4690	0.04

**Figure 4:**
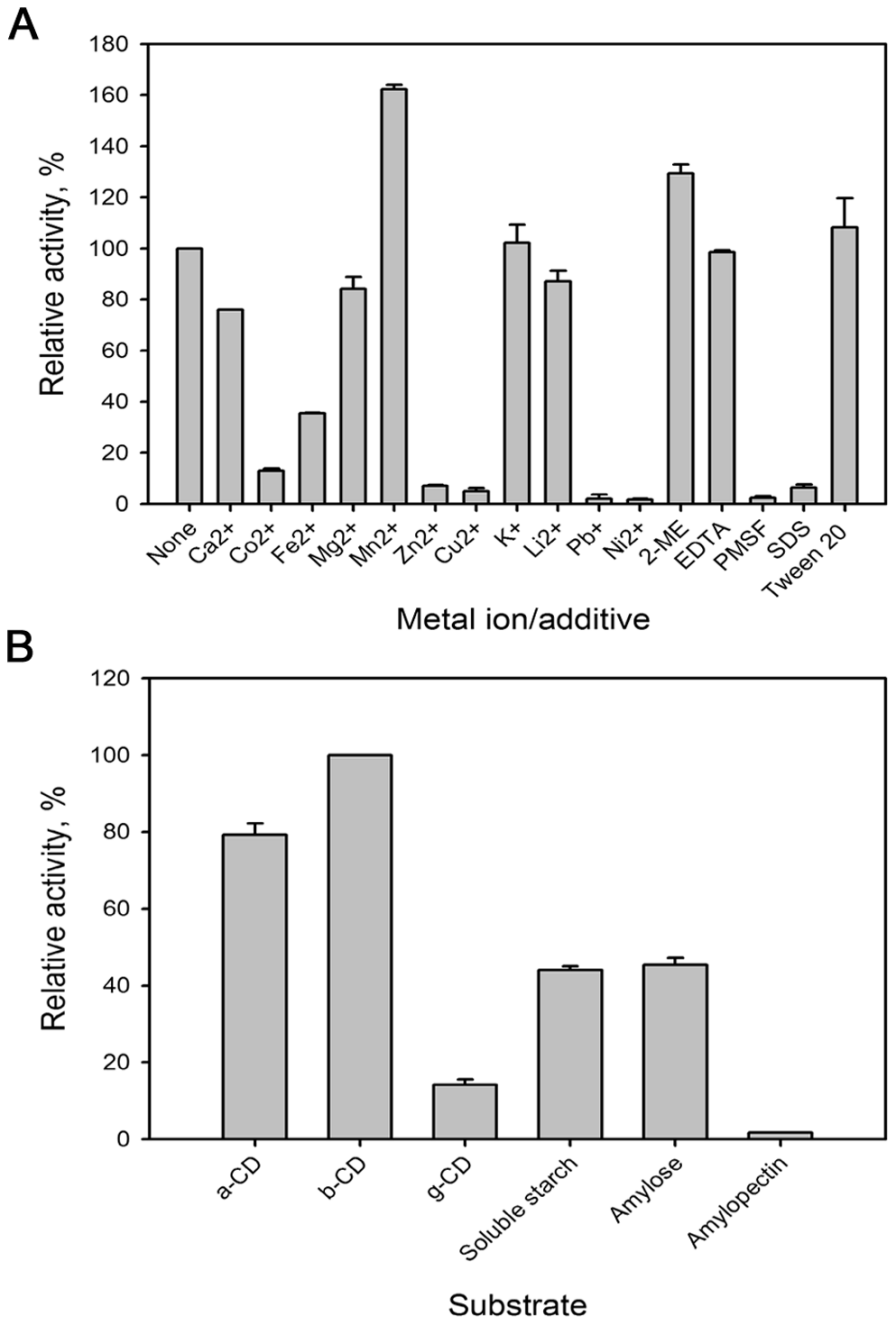
The influence of modulators and substrate specificity of MAG1. (A) The effects of metal ions and additives on MAG1 activity; (B) The action of MAG1 on different substrates. The percentage of activity in the presence of metal ions or additives was calculated relative to the MAG1 activity without any additional modulators. The percentage of activity toward various substrates was calculated relative to MAG1 activity toward β-CD. The error bars represent the standard deviations of triplicate experiments.

The effects of metal ions and additives on MAG1 activity are shown in Figure 4A. The catalytic activity of MAG1 was strongly enhanced by Mn^2+^, with a relative activity of 162%. Although stimulation by Mn^2+^ has commonly been shown for α-amylases, enhancement of the activity of several maltogenic amylases was also reported [22], [24]. However, most maltogenic amylases were inhibited by Mn^2+^ ions. The activity enhancement by Mn^2+^ occurred due to its known protective function against oxidation [25]. MAG1 activity was also enhanced by 2-ME with relative activity of 129%. The enhancement shows the role of the thiol group in inhibiting the oxidation of the free sulfhydryl group-containing amino acids of the protein.

MAG1 activity was not affected by EDTA, suggesting that no metal ions are required for its activity. The result complemented that on maltogenic amylase from *Geobacillus thermoleovorans* [24]. The activity was also not changed by K^+^, and MAG1 was stable in the presence of a surfactant, Tween 20. Slight activity reductions were observed in the presence of Li^2+^, Mg^2+^ and Ca^2+^ (relative activities of 87%, 84% and 76%, respectively). The enzyme activity was significantly inhibited by most heavy metal ions, including Fe^2+^, Co^2+^, Zn^2+^, Cu^2+^, Pb^2+^ and Ni^2+^ (relative activities of 36%, 13%, 7%, 5%, 2% and 2%, respectively). Enzyme inactivation occurred due to the oxidative modifications upon the strong binding of the cations to the sulfhydryl group-containing amino acids [24], [26]. MAG1 activity was also strongly inhibited by PMSF and SDS (relative activities of 2% and 6%, respectively), similarly to maltogenic amylase from *Geobacillus caldoxylosilyticus* TK4 and *Bacillus* sp.WPD616 [22], [27].

### Substrate specificity and kinetic analysis

MAG1 exhibited hydrolytic activity towards a broad range of substrates. The highest MAG1 hydrolytic activity was observed towards β-CD, which was followed by α-CD, amylose, soluble starch and γ-CD (Figure 4B). However, MAG1 only slightly hydrolysed amylopectin. As shown in the multiple sequence alignment (Figure 1), the ‘MPKLN’ sequence is conserved in MAG1; this region is responsible for the modification of the active site geometry for more suitable occupation by the CD molecule [8]. In addition, gel filtration chromatography analysis showed the presence of dimeric forms of the MAG1 molecule. The dimeric form of maltogenic amylase has been reported to have a higher affinity towards β-CD because of the narrower shape of the active site pocket [28]. The shallow active site was shaped by the intertwined N-terminal domains of two monomers that form a dimeric structure [8]. The deletion of the N-terminal domain of maltogenic amylase from *Geobacillus thermoleovorans* caused the dimeric molecule to monomerise and reduced its affinity and efficiency for catalysing β-CD [24]. The monomeric form of maltogenic amylase has a wider active site for the occupation of larger molecules such as starch.

The kinetic analysis showed that the enzyme has a higher affinity for β-CD than for starch, as indicated by the lower K_m_ value (Table 2). A higher turnover number, or K_cat,_ was also observed for β-CD, which implied that more β-CD was hydrolysed per second under optimal conditions compared with soluble starch. Moreover, MAG1 hydrolysed β-CD more efficiently than starch, as demonstrated by the higher enzyme efficiency value or K_cat_/K_m_. These results were consistent with other reported maltogenic amylases, which exhibited a higher substrate affinity towards β-CD [5], [21], [29]. Maltogenic amylases were more likely to cleave α-(1,4)-glycosidic bonds more efficiently compared with α-(1,6) glycosidic bonds [30]. Therefore, the β-CD molecule, which consists of glucose units that are linked by α-(1,4)-glycosidic bonds, was efficiently hydrolysed by MAG1. The hydrolysis of starch by MAG1 was presumably selective for amylose but not amylopectin, as MAG1 scarcely hydrolysed amylopectin. The groove shape of the active site can only accommodate the linear part of the starch (amylose), whereas the branching part of the starch that consists of α-(1,6)-glycosidic bonds (amylopectin) would not easily reach the catalytic residues [31]. This selective degradation is commonly displayed by CD-degrading enzymes [32], and it has been used efficiently in producing amylose-free starch with a low retrogradation rate [33].

**Table 2:** MAG1 kinetic parameters for β-CD and soluble starch.

Substrate	V_max_ (µmol mg^−1^ min^−1^)	K_m_ (mg ml^−1^)	K_cat_ (s^−1^)	K_cat_/K_m_ (ml mg^−1^ s^−1^)
β-CD	667	1.27	751	593
Soluble starch	526	1.74	593	341

### Hydrolysis product specificity of MAG1

**Figure 5:**
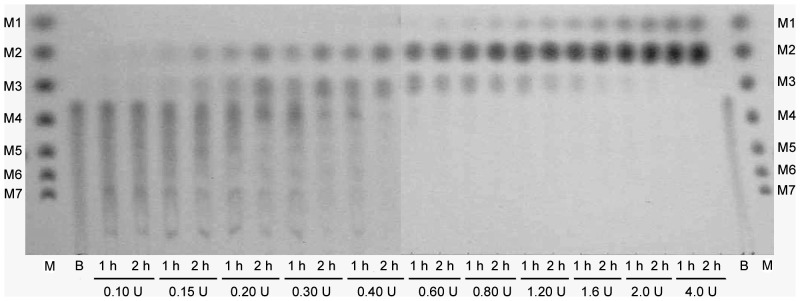
The separation of hydrolysis products by TLC. β-CD was hydrolysed using different enzyme concentrations for 2 hours. M: standard malto-oligosaccharides, M1, glucose; M2, maltose; M3, maltotriose; M4, maltotetraose; M5, maltopentaose; M6, maltohexaose; and M7, maltoheptaose, B: β-CD standard, 1 h: sample collected after 1 hour, 2 h: sample collected after 2 hours, U: units of MAG1 per mg β-CD.

**Figure 6:**
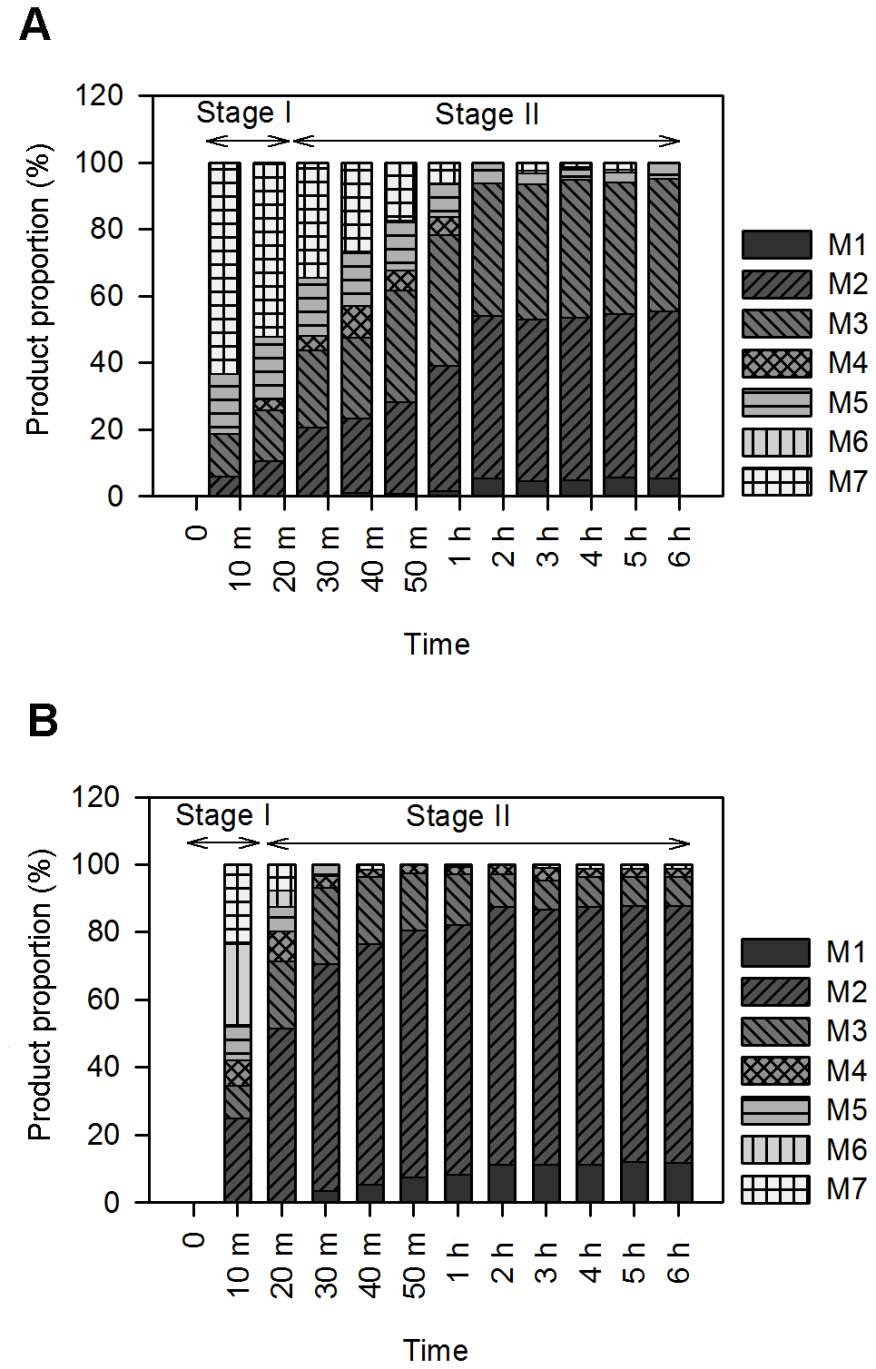
Time course for the hydrolysis of β-CD by MAG1. The different product selectivity values were observed when different concentrations of the enzyme were used; (A) 0.4 U MAG1/mg β-CD and (B) 1.6 U MAG1/mg β-CD. Hydrolysis products consisted of M1, glucose; M2, maltose; M3, maltotriose; M4, maltotetraose; M5, maltopentaose; M6, maltohexaose; and M7, maltoheptaose. The product proportion was presented as a mean percentage from three triplicate experiments. The standard deviations were less than 0.6%.

β-CD was incubated with different concentrations of MAG1 to determine the hydrolysis product specificity. According to the TLC analysis (Figure 5), the final end products of β-CD hydrolysis varied with changes in enzyme concentration. A time course hydrolysis was performed for two concentrations of MAG1, 0.4 U/mg β-CD and 1.6 U/mg β-CD, which represented the low and high enzyme concentrations, respectively. Two stages of hydrolysis were identified in both concentrations, as presented in Figure 6. The first stage involved the linearisation of the β-CD ring, which produced M7 as a major product. The ring-opening reaction involved the cleavage of one α-(1,4)-glycosidic linkage of β-CD molecules. A similar catalytic property of the ring-opening reaction was also reported in several amylases [23], [34]. The linear M7 was also eventually hydrolysed resulting in the formation of other shorter linear malto-oligosaccharides at this stage. The second stage involved the hydrolysis of M7 to produce shorter malto-oligosaccharides. Stage I persisted for 20 min when 0.4 U MAG1/mg β-CD was used, and M2 and M3 were produced as the final end products (Figure 6A). In contrast, Stage I was shortened to 10 min when 1.6 U MAG1/mg β-CD was used, and M1 and M2 were produced as the final end products (Figure 6B). The reactions for both enzyme concentrations were completed after 2 hours, as observed from the stable level of the final end products.

Typically, most reported maltogenic amylases primarily produced M2 and M1 by β-CD hydrolysis. Table 3 summarises the hydrolysis products of specific substrates by various sources of maltogenic amylases, α-amylases and other CD-degrading enzymes. Neopullulanase and cyclomaltodextrinase shared similar product specificity with maltogenic amylases. This finding is explained by the indistinguishable active site shape, structure and catalytic properties of these enzymes [35]. Conversely, some α-amylases produced malto-oligosaccharides, maltotriose and maltose as major final-end products of starch hydrolysis. Interestingly, MAG1 exhibited product specificities for both maltogenic amylases and α-amylases. As shown in Figure 5, malto-oligosaccharides longer than M3 were the predominant products at a low enzyme unit concentration. As the enzyme concentration increased, M2 and M3 became the major final end products. At higher enzyme concentrations, the final end products were M1 and M2. This unique characteristic has never been reported for any other maltogenic amylases. It was suggested that at a limited enzyme concentration, the active sites of the enzyme were fully occupied by β-CD molecules. Malto-oligosaccharides, which were the products of β-CD hydrolysis, were prevented from entering the active site in the presence of excess β-CD molecules because maltogenic amylase and other CD-degrading enzymes have higher substrate specificities and stronger affinities for β-CD compared with malto-oligosaccharides [32], [34]. Conversely, in the presence of excess enzyme, more active sites were available for occupation by malto-oligosaccharides in addition to β-CD. Thus, malto-oligosaccharides were concurrently hydrolysed, producing smaller sugar polymers as the final end products.

**Table 3:** Product variations in response to the hydrolysis of various substrates by different amylolytic enzymes.

Enzyme	Source	Substrate	Hydrolytic products	Reference
Maltogenic amylase	*Bacillus lehensis* G1	β-cyclodextrin	Glucose, maltose, maltotriose and malto-oligosaccharides	Current study
Maltogenic amylase	*Bacillus* sp. US149	β-cyclodextrin	Glucose, maltose	[21]
Maltogenic amylase	*Geobacillus caldoxylosilyticus* TK4	β-cyclodextrin	Glucose, maltose	[22]
Maltogenic amylase	*Thermofilum pendens*	γ-cyclodextrin	Glucose, maltose	[47]
Maltogenic amylase	*Staphylothermus marinus*	β-cyclodextrin	Glucose, maltose	[23]
Maltogenic amylase	*Lactobacillus gasseri* ATCC33323	β-cyclodextrin	Glucose, maltose	[48]
Cyclomaltodextrinase	Soil isolated gene	α, β and γ-cyclodextrin	Glucose and malto-oligosaccharides	[49]
Cyclomaltodextrinase	*Clostridium thermohydrosulfuricum* 39E	β-cyclodextrin	Glucose, maltose	[50]
Neopullulanase	*Bacillus stearothermophilus* TRS40	Amylose	Maltose	[32]
α-amylase	*Streptococcus bovis* 148	Starch	Maltotriose	[51]
α –amylase	*Saccharopolyspora* sp. A9	Starch	Glucose, maltose and maltotriose	[52]
α -amylase	*Chromohalobacter* sp. TVSP 101	Starch	Maltotetraose, maltotriose, maltose and glucose	[53]
α –amylase	*Bacillus* sp. KR11	Starch	Maltotriose	[54]
α –amylase	*Marinobacter* sp. EMB8	Starch	Maltose, maltotriose, maltotetraose	[43]

The specific product of β-CD hydrolysis by MAG1 could be determined and achieved by manipulating the enzyme unit concentration. The absence of glucose at low enzyme concentrations provides an added value to the hydrolysis products of MAG1 and a simplified downstream processing, which requires the separation of glucose from other malto-oligosaccharides. The presence of glucose in malto-oligosaccharide products is undesirable in food industry applications [36]. The high maltotriose-producing property of MAG1 has potential applications in the production of maltotriose-containing syrup. Maltotriose is known for its hygroscopic properties, mild sweetness and usefulness in preventing the retrogradation of starch in the food industry [37].

### Malto-oligosaccharide synthesis by MAG1

**Figure 7:**
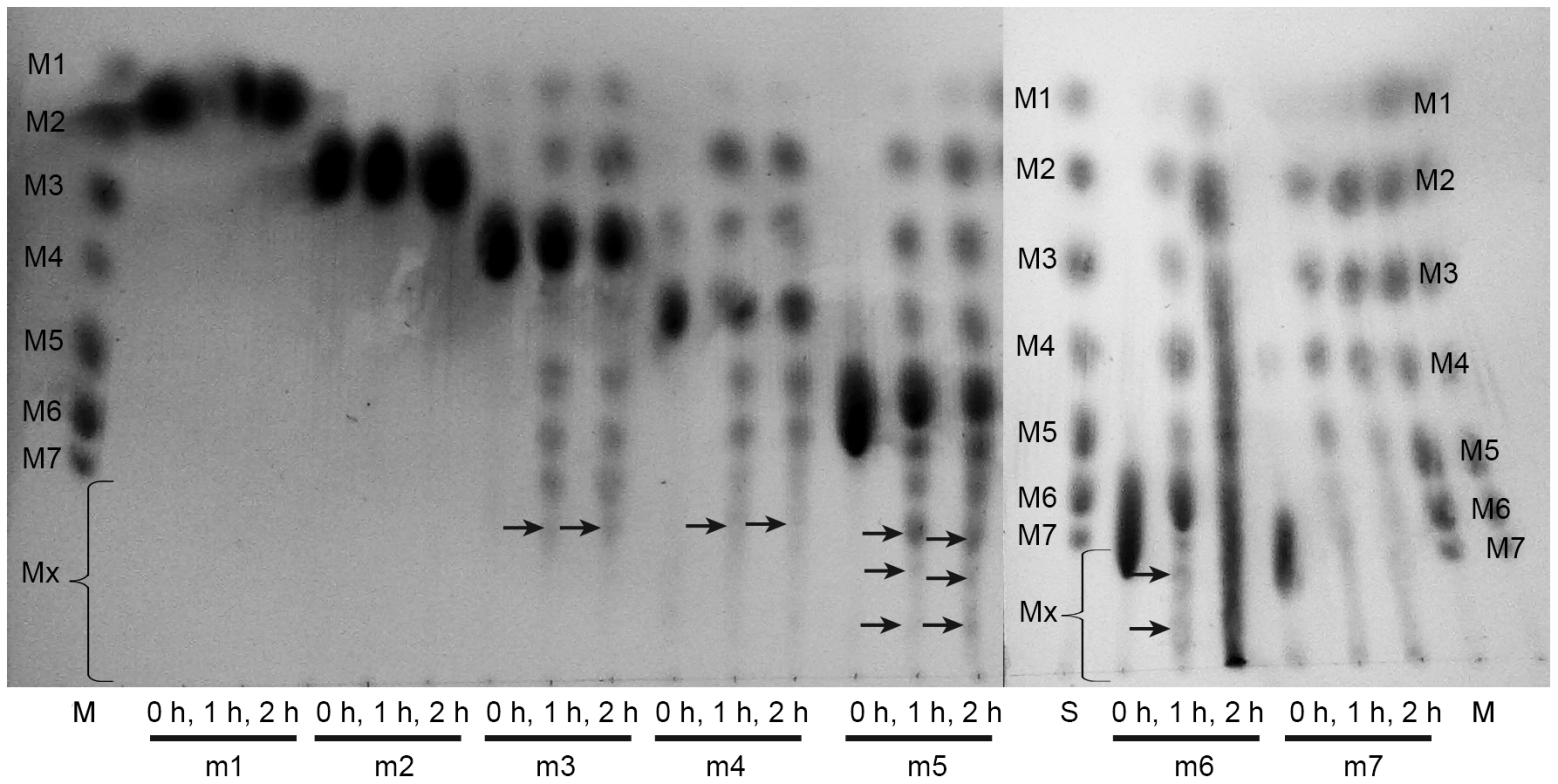
The separation of the transglycosylation products by TLC. The excess quantity of malto-oligosaccharides (5% w/v) was incubated with 2.5 U of MAG1 for 2 hours. M: standard malto-oligosaccharides, m: malto-oligosaccharides that were added as donor and acceptor molecules for transglycosylation, 0 h: sample collected at 0 hours, 1 h: sample collected after 1 hour, 2 h: sample collected after 2 hours, Mx: malto-oligosaccharides longer than M7. The arrows indicate malto-oligosaccharide spots longer than M7.

Maltogenic amylases have been shown to catalyse the transglycosylation reaction, in addition to their hydrolytic action on multiple substrates. To investigate MAG1 transglycosylation activity, the enzyme was incubated with 5% (w/v) (various) malto-oligosaccharides. These malto-oligosaccharides acted as both donor and acceptor molecules for transglycosylation. Based on the TLC analysis, MAG1 catalysed the transglycosylation for M3 and longer malto-oligosaccharides but not the smaller molecules (Figure 7). This result indicated that M3 and longer malto-oligosaccharides were good donor molecules for transglycosylation.

MAG1 synthesised malto-oligosaccharides of various lengths, some of which were even longer than M7, as presented in Figure 7. However, smaller sugar polymers were also detected, which implies that the elongated products were hydrolysed by the same enzyme. Hydrolysis is a common disadvantage of using glycosyl hydrolases (GHs) for oligosaccharide synthesis in comparison with glycosyl transferases [11]. Despite this drawback, GHs were still preferred because of their availability, stability, ease of handling and activity on inexpensive simple substrates without requiring cofactors [2], [38].

### Optimisation of M3 transglycosylation by MAG1

The inevitable hydrolysis activity by GHs is undesirable because it adversely affects the yield of the oligosaccharides produced. However, the thermodynamic equilibrium can be shifted towards synthesis by optimising the reaction conditions to suppress hydrolysis. In the present study, M3 was selected as both the donor and acceptor molecule for transglycosylation because it was the simplest sugar that could undergo elongation by MAG1 (Figure 7), and its transglycosylation products were easily detected and quantified by HPLC and TLC. The transglycosylation activity was evaluated by measuring the proportion of malto-oligosaccharides with a polymerisation degree higher than M3, and the hydrolysis activity was evaluated by measuring the proportions of M1 and M2 in the final reaction products.

**Figure 8:**
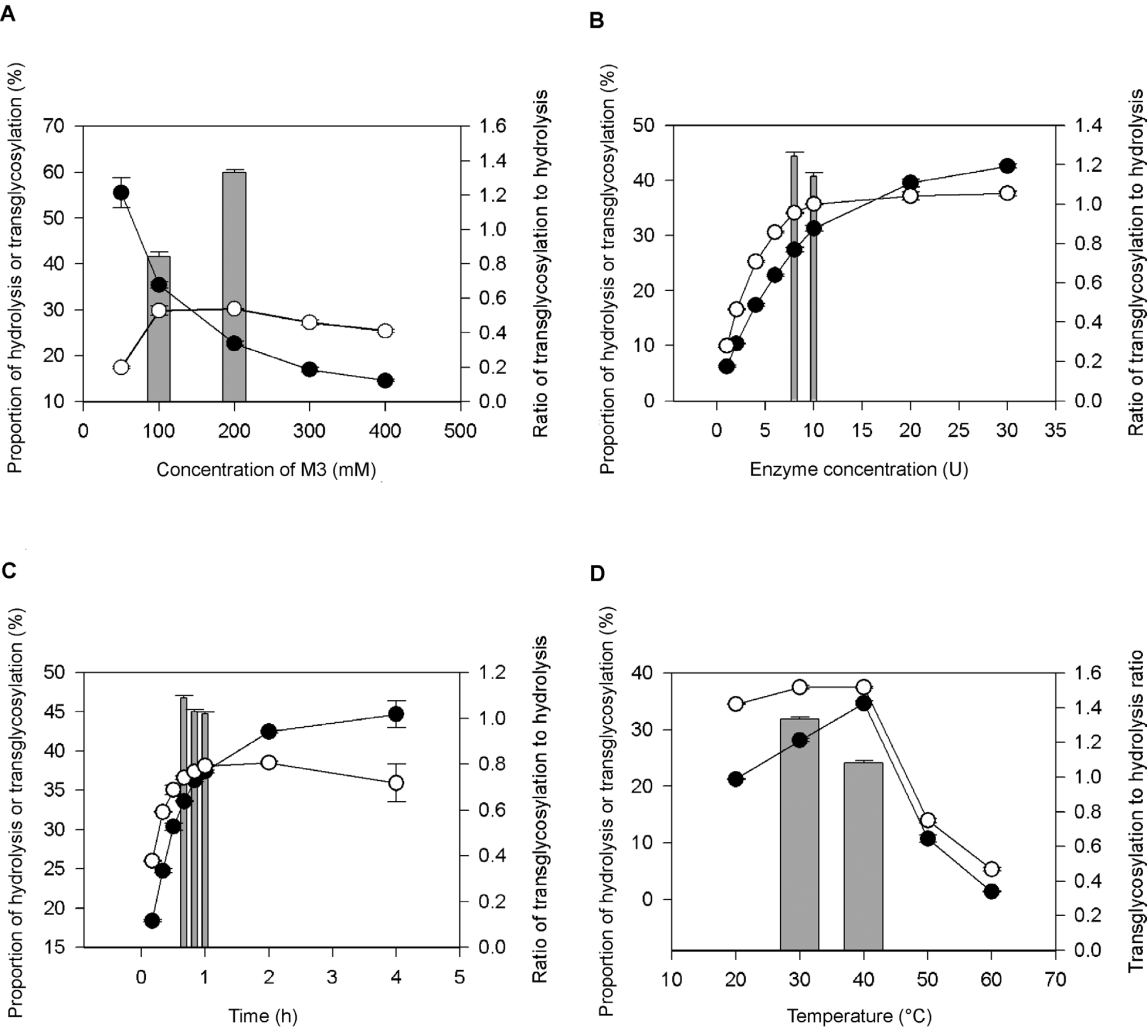
The optimisation of the transglycosylation reaction. The effects of (A) M3 concentration, (B) enzyme concentration, (C) time and (D) temperature on the proportion of hydrolysis or transglycosylation are shown. White circles (○) designate the transglycosylation proportion, black circles (•) designate the hydrolysis proportion and bar charts designate the ratio of transglycosylation to hydrolysis. Error bars represents the standard deviations of triplicate experiments.

Figure 8 demonstrates that the reaction equilibrium can be shifted to favour hydrolysis or transglycosylation by manipulating the reaction conditions. Four primary factors were shown to influence the reaction equilibrium greatly. First, the transglycosylation became dominant at an M3 concentration of 200 mM and higher, and hydrolysis was dominant at lower concentrations (Figure 8A). Water is the competing nucleophile in the transglycosylation reaction [38]. Therefore, the high substrate concentration limited water availability, thereby reducing the hydrolytic activity of MAG1. Moreover, it provided the reaction with more acceptor molecules to increase the synthesis rate [39]. The saturated substrate concentration was achieved at 200 mM. The further addition of M3 did not increase the proportion of transglycosylation. Therefore, 200 mM M3 was found to be the best concentration for transglycosylation and was used in the following reaction optimisations. Second, transglycosylation was dominant at an enzyme concentration of 10 U and lower (Figure 8B). Adding more enzyme would only shift the reaction equilibrium toward hydrolysis because more active sites would be available to accommodate the elongated M3 products. Although the transglycosylation proportion was slightly higher for 10 U MAG1 than 8 U, the ratio of transglycosylation to hydrolysis was lower. Therefore, 8 U of MAG1 was found to be the best concentration for maintaining high transglycosylation with relatively low hydrolysis activity.

Third, when malto-oligosaccharides were simultaneously synthesised and hydrolysed by MAG1, the harvesting time greatly influenced the proportion of competing reactions. As shown in Figure 8C, hydrolysis started to compete with transglycosylation at 1 hour and dominated when the incubation time was prolonged. Therefore, 40 min was chosen as the optimal time for harvesting when the hydrolysis proportion was still low and the transglycosylation was reaching its maximum yield. Moreover, the transglycosylation-to-hydrolysis ratio was the highest at this point. Fourth, the optimum temperature for hydrolysis by MAG1 was reported to be 40°C (Figure 3A) and the hydrolysis dropped significantly at 30°C. However, transglycosylation occurred to the same extent at both temperatures, resulting in a higher transglycosylation-to-hydrolysis ratio at 30°C compared with 40°C (Figure 8D). Bond formation releases energy, and conversely, bond breaking requires energy [40]. Therefore, hydrolysis that involved the breakage of glycosidic bonds was most effective at a higher temperature, whereas transglycosylation was equally efficient at a lower temperature.

Taken together, all four optimised conditions contributed to a transglycosylation proportion of approximately 38% of the total reaction products. The transglycosylation yield obtained in this study was higher than the reported transglycosylation yield of maltotriose by maltogenic amylase from *Bacillus stearothermophilus* ET1, which was 29.4% [30]. Transglycosylation of acarbose by maltogenic amylases has also been reported, but none of the studies provided the yield of the transglycosylation products [4], [5]. However, β-galactosidases exhibited transglycosylation yields between 30% and 40% to produce galacto-oligosaccharides from lactose. The galacto-oligosaccharide production reportedly did not exceed 50% [41].

### Hydrolysis suppression by organic solvent

The water activity level is largely responsible for shifting the thermodynamic equilibrium of a reaction toward hydrolysis or transglycosylation. As shown in Figure 8A, a high M3 concentration, which indicated low water activity, greatly suppressed hydrolysis. The retardation of water activity could promote a high reaction rate in hydrophobic compounds, thus increasing transglycosylation. The water activity was further reduced by incorporating a water-miscible organic solvent into the reaction mixture. As indicated in Table 4, the addition of various organic solvents reduced the hydrolysis proportion giving rise to the transglycosylation-to-hydrolysis ratio. The addition of 10% ethanol, 2-propanol and dimethyl sulphoxide (DMSO) increased the transglycosylation-to-hydrolysis ratio from 1.29 (control) to 1.56, 1.86 and 2.11, respectively. The addition of 20% ethanol and DMSO led to a further increase in the ratio by 2.15. However, the addition of 20% 2-propanol and the further addition of ethanol and DMSO caused a total loss of activity because of the structural changes.

**Table 4:** Effects of organic solvents on the proportion of hydrolysis, transglycosylation and the product ratio.

Solvent	Concentration (%)	Hydrolysis^a^ (%)	Transglycosylation^a^ (%)	Composition of malto-oligosaccharides^a^ (%)				T:H^b^
				M4	M5	M6	M7	
None	-	29.09	37.78	12.20	10.68	6.84	8.06	1.29
Ethanol	10	22.45	35.05	9.41	11.25	7.12	7.28	1.56
	20	17.05	36.67	7.66	14.35	7.53	7.12	2.15
2-Propanol	10	17.57	32.62	7.20	11.33	7.00	7.08	1.86
	20	2.38	3.06	0.00	1.95	0.65	0.47	1.29
DMSO	10	16.28	34.38	7.14	12.69	7.43	7.13	2.11
	20	17.31	37.28	7.79	14.58	7.66	7.25	2.15

a Values shown are the means for triplicate experiments. The values of the standard deviations were below 0.5.

b Transglycosylation-to-hydrolysis ratio (T:H).

The increased transglycosylation-to-hydrolysis ratio occurred because of the decreased hydrolysis activity when an organic solvent was incorporated in the reaction. Hydrolysis was suppressed from 29.09% to 16.28% (Table 4). At a low concentration, organic solvents may exert an inhibitory effect on hydrolysis by displacement of the hydration layer in the catalytic site of the enzyme [42]. Furthermore, the product selectivity for maltopentaose (M5) was improved from 10.68% to 14.35% and 14.58% by the addition of ethanol and DMSO, respectively (Table 4). The hydrolysis of M5 might be inhibited by the organic solvent. Several studies have reported the enhancement of malto-oligosaccharide production by α-amylases from starch [42], [43], galacto-oligosaccharides by β-galactosidase from lactose [12], [44] and cyclodextrin by cyclodextrin glucanotransferase from starch [45] in the presence of an organic solvent. However, little is known about the effects of organic solvents on the production of malto-oligosaccharides by maltogenic amylase. Moreover, this is the first report on the effects of organic solvents relating to the hydrolysis and transglycosylation reactions through maltogenic amylase. The present study showed that the addition of an organic solvent could be used to improve the product selectivity of maltogenic amylase for longer malto-oligosaccharides.

### Malto-oligosaccharides with a high degree of polymerisation

**Figure 9:**
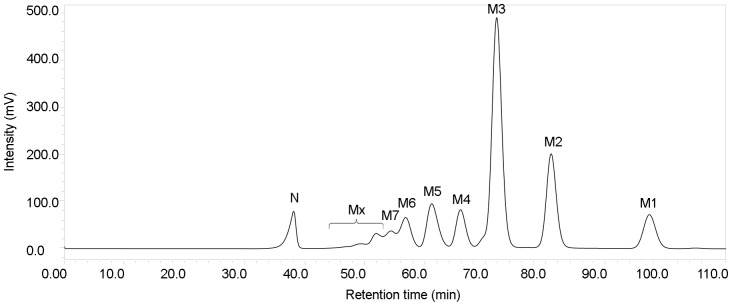
The HPLC separation of the malto-oligosaccharides produced from MAG1 transglycosylation activity on maltotriose. The reaction was performed at the optimum conditions. M1, glucose; M2, maltose; M3, maltotriose; M4, maltotetraose; M5, maltopentaose; M6, maltohexaose; and M7, maltoheptaose, Mx, malto-oligosaccharides longer than M7, and N, background noise.

Enzyme-catalysed glycosidic bond formation in carbohydrate synthesis provides a simple reaction step and avoids the use of extensive protection-deprotection sequences [38]. In recent years, glycosidases have been frequently employed for novel carbohydrate and oligosaccharide syntheses. It is notable that MAG1 was able to produce malto-oligosaccharides from M4 to M7 and longer than M7, and it also used a broad range of acceptors for transglycosylation, such as M3, M4, M5, M6 and M7. As shown in Table 5, the yield of malto-oligosaccharide produced by MAG1 was higher (38%) compared with the yield of oligosaccharides produced by other enzymes, and a relatively low substrate concentration (100 mg/ml) was used to achieve the yield. Moreover, the longest chain of oligosaccharides produced by various enzymes were characterised up to tetra-saccharides. In contrast, MAG1 could synthesise malto-oligosaccharides longer than tetra-saccharides. Longer malto-oligosaccharides were clearly spotted on the TLC plate and were also displayed by the peaks in the HPLC chromatogram (Figures 7 and 9, respectively). This characteristic has never been reported for other maltogenic amylases. Longer oligosaccharides are desirable as prebiotics because they are less fermentable and, therefore, can reach the more distal area of the colon [46]. This finding was an earlier indicator that MAG1 was a promising synthetic tool for producing malto-oligosaccharides and novel carbohydrates.

**Table 5:** Comparison of oligosaccharide production from various enzymes.

Enzyme	Source	Substrate	Initial substrate concentration (mg/ml)	Yield (%)	Length of oligosaccharides	Reference
Maltogenic amylase	*Bacillus lehensis* G1	Maltotriose	100	38.0	Tetra- to hepta-saccharides and oligosaccharides longer than 7	Current study
Maltogenic amylase	*Bacillus stearothermophilus* ET1	Maltotriose	100	29.4	Not characterised	[30]
Neopullulanase	*Bacillus stearothermophilus* TRS40	Maltotriose	100	17.1	Di- to tri-saccharides and oligosaccharides longer than 4	[55]
α-Glucosidase	*Xanthophyllomyces dendrorhous*	Maltose	200	26.9	Tri- and tetra-saccharides	[56]
β-Glucosidase	*Pyrococcus furiosus*	Lactose	700	40.0	Tri- and tetra-saccharides	[2]
β-Galactosidase	*Sulfolobus solfataricus*	Lactose	600	37.0	Tri- and tetra-saccharides	[57]

## Conclusions

MAG1 is a good biocatalyst for producing malto-oligosaccharides of various lengths through β-CD hydrolysis or transglycosylation. The optimisation of reaction conditions and the incorporation of water-miscible organic solvent led to the suppression of hydrolysis and shifted the thermodynamic equilibrium toward transglycosylation. The high transglycosylation activity displayed by MAG1 produced malto-oligosaccharides with degrees of polymerisation higher than M7, which demonstrated that MAG1 is a promising candidate for carbohydrate synthesis applications.
